# Supplementary material for: Direct thin-layer agar for bedaquiline-susceptibility testing of Mycobacterium tuberculosis at BSL2 level yields high accuracy in 15 days from sputum processing
Source: J Clin Microbiol. 2025 Mar 3;63(4):e01806-24. doi: 10.1128/jcm.01806-24 (PMC11980359; doi:10.1128/jcm.01806-24)
Supplement: Table S1 — Characteristics, genetic profile, and median BDQ-MIC values for described MTB isolates used in this study, comparing TLA and 7H11 methods. [file jcm.01806-24-s0001.docx]

**Table of Contents**

Supplementary Tables 2

**Supplementary Tables**

**Table S1**. Characteristics, genetic profile, and median BDQ-MIC values for described MTB isolates used in this study, comparing TLA and 7H11 methods.

| **Isolate number** | **Lineage** | **Origin** | ***Rv0678*** | ***atpE*** | **Final confidence grading WHO** | **TLA MIC median** | **7H11 MIC** |
| --- | --- | --- | --- | --- | --- | --- | --- |
| 1999-01856 | Unknown | Clinical | Unknown | unknown |  | 0.25 | 0.25 |
| 2012-00594 | Unknown | Clinical | Unknown | unknown |  | 0.06 | 0.125 |
| 2014-01618 | Unknown | Clinical | Unknown | unknown |  | 1 | 1 |
| 2015-02105 | L4 | Clinical | WT | WT |  | 0.06 | 0.06 |
| 2015-02116 | L2 | Clinical | WT | WT |  | 0.03 | 0.03 |
| 2015-02119 | L2 | Clinical | WT | WT |  | 0.06 | 0.06 |
| 2015-02124 | L2 | Clinical | WT | WT |  | 0.06 | 0.03 |
| 2015-02137 | L2 | Clinical | WT | WT |  | 0.06 | 0.06 |
| 2015-02146 | L2 | Clinical | WT | WT |  | 0.03 | 0.03 |
| 2015-02147 | L2 | Clinical | p.Tyr157Cys | WT | Not present in catalogue 2^nd^ ed | 0.25 | 0.25 |
| 2015-02150 | L2 | Clinical | WT | WT |  | 0.06 | 0.03 |
| 2015-02161 | L4 | Clinical | WT | WT |  | 0.06 | 0.03 |
| 2018-00082 | L1 | Clinical | WT | WT |  | 0.06 | 0.125 |
| 2018-00089 | L3 | Clinical | WT | WT |  | 0.06 | 0.06 |
| 2018-00090 | L3 | Clinical | WT | WT |  | 0.06 | 0.125 |
| 2018-00092 | L4 | Clinical | c.692C>T | WT | Not present in catalogue 2^nd^ ed | 0.06 | 0.125 |
| 2018-00094 | L4 | Clinical | WT | WT |  | 0.06 | 0.03 |
| 2018-00102 | L1 | Clinical | WT | WT |  | 0.06 | 0.06 |
| 2019-00870 | L2 | Clinical | WT | WT |  | 0.06 | 0.06 |
| 2019-00875 | L2 | Clinical | p.Arg89Leu | WT | Uncertain significance | 0.5 | 0.5 |
| 2019-00918 | L2 | Clinical | WT | WT |  | 0.06 | 0.03 |
| 2013-00482 | L4 | In vitro selected | p.Arg135Trp | WT | Uncertain significance | 0.5 | 1 |
| 2013-02484 | L1 | In vitro selected | p.Tyr92STP | WT | Assoc w R - Interim | 1 | 1 |
| 2013-02485 | L1 | In vitro selected | p.Glu138Gly-p.Met139Leu | WT | Not present in catalogue 2^nd^ ed | 0.5 | 1 |
| 2014-01612 | L1 | In vitro selected | p.Ser63Arg | WT | Uncertain significance | 1 | 1 |
| 2014-01618 | L4 | In vitro selected | p.Arg135Trp | WT | Uncertain significance | 0.5 | 1 |
| 2014-02967 | L2 | In vitro selected | WT | p.Ala63Pro | Assoc w R - Interim | 1 | 1 |
| 2014-02973 | L2 | In vitro selected | WT | p.Glu61Asp | Assoc w R - Interim | 0.5 | 0.5 |
| 2014-02988 | L2 | In vitro selected | p.Gly78Val | WT | Not present in catalogue 2^nd^ ed | 0.25 | 0.5 |
| 2014-03045 | L2 | In vitro selected | WT | p.Ala63Pro | Assoc w R - Interim | 2 | 2 |
| 2015-00554 | L4 | In vitro selected | WT | p.Asp28Gly | Assoc w R - Interim | 0.5 | 0.25 |
